# MicroRNAs and long non-coding RNAs in pancreatic cancer: From epigenetics to potential clinical applications

**DOI:** 10.1016/j.tranon.2022.101579

**Published:** 2022-11-01

**Authors:** Luis Alberto Bravo-Vázquez, Natalia Frías-Reid, Ana Gabriela Ramos-Delgado, Sofía Madeline Osorio-Pérez, Hania Ruth Zlotnik-Chávez, Surajit Pathak, Antara Banerjee, Anindya Bandyopadhyay, Asim K. Duttaroy, Sujay Paul

**Affiliations:** a School of Engineering and Sciences, Tecnologico de Monterrey, Campus Queretaro, *Av.* Epigmenio Gonzalez, No. 500 Fracc. San Pablo, Queretaro 76130, Mexico; b Department of Medical Biotechnology, Faculty of Allied Health Sciences, Chettinad Academy of Research and Education (CARE), Chettinad Hospital and Research Institute (CHRI), Chennai, India; c International Rice Research Institute, Manila 4031, Philippines; d Reliance Industries Ltd., Navi Mumbai 400701, India; e Department of Nutrition, Institute of Basic Medical Sciences, Faculty of Medicine, University of Oslo, POB 1046, Blindern, Oslo, Norway

**Keywords:** Pancreatic cancer, MicroRNAs, lncRNAs, Biomarker, Therapeutics

## Abstract

MicroRNAs (miRNAs) and long non-coding RNAs (lncRNAs) are two relevant classes of non-coding RNAs (ncRNAs) that play a pivotal role in a number of molecular processes through different epigenetic regulatory mechanisms of gene expression. As a matter of fact, the altered expression of these types of RNAs leads to the development and progression of a varied range of multifactorial human diseases. Several recent reports elucidated that miRNA and lncRNAs have been implicated in pancreatic cancer (PC). For instance, dysregulation of such ncRNAs has been found to be associated with chemoresistance, apoptosis, autophagy, cell differentiation, tumor suppression, tumor growth, cancer cell proliferation, migration, and invasion in PC. Moreover, several aberrantly expressed miRNAs and lncRNAs have the potential to be used as biomarkers for accurate PC diagnosis. Additionally, miRNAs and lncRNAs are considered as promising clinical targets for PC. Therefore, in this review, we discuss recent experimental evidence regarding the clinical implications of miRNAs and lncRNAs in the pathophysiology of PC, their future potential, as well as the challenges that have arisen in this field of study in order to drive forward the design of ncRNA-based diagnostics and therapeutics for PC.

## Highlights

• Both miRNA and lncRNA expression is dysregulated in pancreatic cancer.
• Altered miRNA and lncRNA expression is related to pancreatic cancer progression.
• miRNAs and lncRNAs affect tumor suppressor genes, oncogenes, and signaling pathways.
• miRNAs and lncRNAs are prospective theragnostic targets for pancreatic cancer.
• Further studies are required to boost the development of ncRNA-based therapeutics.

## Graphical abstract

**Figure:**
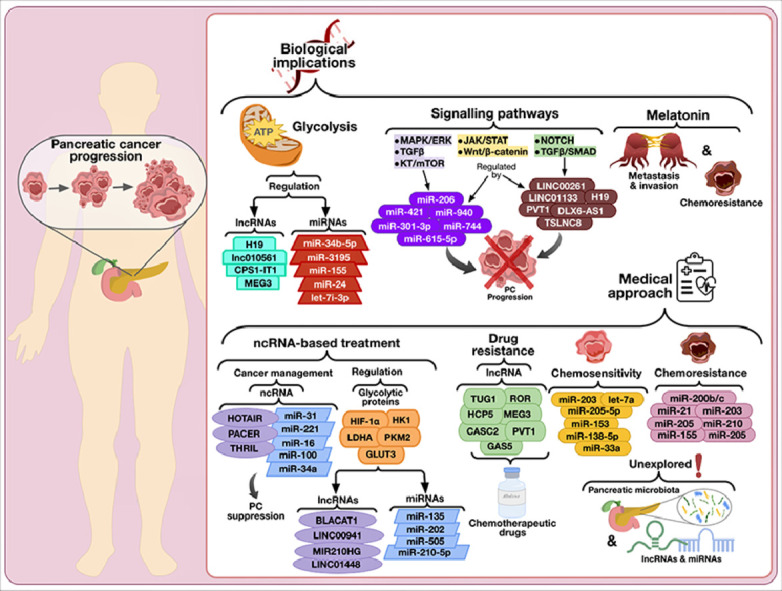

**Abbreviations**
(PFKFB3): 6-Phosphofructo-2-Kinase/Fructose-2,6-Biphosphatase 3
(ABHD11-AS1): ABHD11 antisense RNA 1
(ANRIL): Antisense Non-Coding RNA in the INK4 Locus
(AMF): Autocrine Motility Factor
(ATG5): Autophagy related 5
(AXIN2): Axis Inhibition Protein 2
(BTG2): B-cell Translocation Gene 2
(BLACAT1): Bladder Cancer Associated Transcript 1
(BANCR): BRAF-Activated Non-Protein Coding RNA
(BRCA2): Breast Cancer Type 2 protein
(CASC2): Cancer Susceptibility Candidate 2
(CA 19–9 and CA 199): Carbohydrate antigen 19–9
(CAV1): Caveolin 1
(cfDNA): Cell-free DNA
(CERS6-AS1): Ceramide Synthase 6 Antisense RNA 1
(CRNDE): Colorectal Neoplasia Differentially Expressed
(HOST2): Competing Endogenous lncRNA 2 for microRNA let-7b
(CPS1-IT1): CPS1 Intronic Transcript 1
(CDKN2D): Cyclin Dependent Kinase Inhibitor 2D
(CDKN2A): Cyclin Dependent Kinase inhibitor 2A
(DLEU2L): Deleted In Lymphocytic Leukemia 2 Like
(DNA): Deoxyribonucleic Acid
(DANCR): Differentiation Antagonizing Non-Protein Coding RNA
(ID4): DNA-Binding Protein Inhibition ID-4
(DUXAP8): Double Homeobox A Pseudogene 8
(E2F3): E2F Transcription Factor 3
(PKM2): Enzyme Pyruvate Kinase M2
(EIF5A2): Eukaryotic Translation Initiation Factor 5A2
(ERK2): Extracellular Signal-Regulated Kinases 1
(FEZF1-AS1): FEZ family Zinc Finger 1-Antisense RNA 1
(FGD5-AS1): FGD5 Antisense RNA 1
(FDA): Food and Drug Administration
(FOXA1): Forkhead Box A1
(GATA3-AS1): GATA Binding Protein 3 Antisense RNA 1
(GLUT 1 and GLUT3): Glucose Transporter
(GAPDH): Glyceraldehyde 3-Phosphaste Dehydrogenase
(GOLM1): Golgi Membrane Protein 1
(GAB1): GRB2-Associated Binding Protein 1
(GAS5): Growth Arrest Specific 5
(H19): H19 Imprinted Maternally Expressed Transcript
(H19): H19 maternally expressed transcript
(HK1 and HK2): Hexokinase
(HMGB1): High Mobility Group Box 1
(HCP5): HLA Complex P5
(HOTAIR): HOX Transcript Antisense RNA
(HOTTIP): HOXA Distal Transcript Antisense RNA
(HIF-1α): Hypoxia Inducible Factor 1-Alpha
(HIF1A-AS1): Hypoxia Inducible Factor 1-Alpha Antisense RNA 1
(HIF-1α): Hypoxia-Inducible Factor 1-Alpha
(ING5): Inhibitor of Growth Protein 5
(JAK): Janus Kinase
(KRAS): Kirsten Rat Sarcoma Virus
(KFL12): Kruppel-like Factor 12
(LDHA): Lactate Dehydrogenase A
(LRP6): LDL Receptor Related Protein 6
(ELAVL1): Like RNA Binding Protein 1
(LIN28B): LIN-28 Homolog B
(LINC01111, LINC00671, LINC00857, LINC01094): Long Intergenic Non-Protein Coding RNA
(lncRNAs): Long non-coding RNAs
(MEG3): Maternally Expressed Gene 3
(MMP16): Matrix Metallopeptidase 15
(mRNA): Messenger RNA
(MALAT-1): Metastasis Associated Lung Adenocarcinoma Transcript 1
(miRNAs): MicroRNAs
(MAPK): Mitogen Activated Protein Kinase
(MEK): Mitogen/extracellular Signal-related Kinase
(MEK2): Dual Specificity Mitogen-Activated Protein Kinase 2
(MAP3K10): Mitogen-Activated Protein Kinase 10
(SMAD4): Mother Against Decapentaplegic Homolog 4
(MEF2C): Myocyte Enhancer Factor 2C
(NORAD): Non-coding RNA Activated by DNA Damage
(ncRNAs): Non-coding RNAs
(NOTCH1): Notch homolog 1
(NF-κB): Nuclear Factor Kappa B
(PC): Pancreatic Cancer
(PDAC): Pancreatic Ductal Adenocarcinoma
(PanIN): Pancreatic Intraepithelial Neoplasia
(PARP): Poly-ADP-ribose Polymerase
(PTEN): Phosphatase and Tensin Homolog
(PGI): Phosphoglucose Isomerase
(PI3K): Phosphoinositide 3-Kinases
(PVT1): Plasmacytoma Variant Translocation 1
(AKT): Protein Kinase B
(PIM1 and PIM2): Proto-Oncogene Serine/Threonine-Protein Kinase
(PACER): PTGS2 Antisense NFKB1 Complex-Mediated Expression Regulator RNA
(RAF1): RAF-1 Proto-Oncogene, Serine/Threonine Kinase
(Ras): Rat Sarcoma Virus
(qRT-PCR): Real-Time Quantitative Reverse Transcription PCR
(ROR): Retinoic Acid-Related Orphan Receptors
(NOB1): RNA-Binding Protein NOB1
(SAGE): Serial Analysis of Gene Expression
(TOR): Serine/Threonine Protein Kinase
(STAT): Signal Transducer and Activator of Transcription
(SNHG7, SNHG12 and SNHG8): Small Nucleolar RNA Host Gene
(SOX9): SRY-Box Transcription Factor 9
(STARD13): StAR Related Lipid Transfer Domain Containing 13
(TUG1): Taurin Up-Regulated 1
(TEX10): Testis-Expressed Protein 10
(THRIL): TNF and HNRNPL Related Immunoregulatory long non-coding RNA
(TRIAP1): TP53-Regulated Inhibition of Apoptosis 1
(TP73-AS1): TP73 Antisense RNA 1
(TCF4): Transcription Factor 4
(TGFBR3): Transforming Growth Factor Beta Receptor 3
(TGF-β): Transforming Growth Factor Beta
(TRAF6): Tumor Necrosis Factor Receptor-Associated Factor 6
(TP53): Tumor Protein P53
(TP53INP1): Tumor Protein p53-Inducible Nuclear Protein 1
(TSLNC8): Tumor Suppressive lncRNA on Chromosome 8p12
(YWHGA): Tyrosine 3-Monooxygenase/Tryptophan 5-Monooxygenase Activation Protein Zeta
(YB1): Y-Box Binding Protein 1

## Introduction

Pancreatic cancer (PC) is considered the 12th most prevalent cancer with the 7th highest death rate worldwide [1], [2], [3]. According to the literature, in 2030, PC will have the second-highest cancer mortality rate around the world [4], and by 2040 the total worldwide incidence of PC will have increased by 30% [1]. One of the main symptoms that is significantly linked with PC is jaundice [5]; however, early diagnosis and prevention of PC represent two major concerns since patients hardly manifest symptoms, and there is still a lack of specific markers for the accurate detection of PC tumors [6]. Besides, many PC symptoms such as abdominal pain, diarrhea, constipation, and vomiting are also associated with other gastrointestinal diseases [5]. Consequently, most PC diagnoses occur only at an advanced stage [7], and hence novel biomarkers could be prospective tools for the early and precise diagnosis of this type of cancer [8], [9], [10], [11]. In addition, a number of studies have demonstrated that PC patients suffer from depression, anxiety, fatigue, sleep problems, and decreased quality of life; some of these problems are also attributed to the caregivers of such patients [12], [13], [14].

It is worth mentioning that the genetic background of each individual has a significant impact on the tendency of developing PC [15], [16], [17], [18]. In fact, genetic mutations in several proteins, such as CDKN2A, TP53, SMAD4, KRAS, BRCA2, produce abnormalities in the ductal cells of the pancreas, thus triggering the growth of papillary-like structures that can be transformed into a non-invasive microscopic preneoplastic lesion called pancreatic intraepithelial neoplasia (PanIN) [2,[19], [20], [21], [22]]. PanIN is a well-defined precursor of PC that might play a significant role in the progression of pancreatitis and can damage the cell repair cycles leading to the propagation of the neoplastic lesion process [2,23]. PanIN is divided into four grades based on the degree of both architectural and cytological alterations in pancreatic ducts: PanIN-1A (lowest grade), PanIN-1B and PanIN-2 (intermediate grade), and PanIN 3 (highest grade) [24].

Apart from genetic modifications, epigenetic factors, e.g., DNA methylation, RNA methylation, histone modifications, and non-coding RNAs (ncRNAs), promote PC initiation and progression [25], [26], [27]. DNA methylation is a DNA methyltransferase-mediated process that modifies cytosine residues and alters gene expression by adding a methyl side group, creating 5-methylcytosines [28]. Aberrant hypermethylation and hypomethylation have been shown to predispose patients to PC. Multiple genes show a hypomethylation status in PC, while hypermethylation is usually implicated in the downregulation of tumor suppressor genes, which leads to PC development [29]. Similarly, it has been noticed that the methylation of diverse microRNAs (miRNAs) and long non-coding RNAs (lncRNAs), such as miR‑29b‑3p, LINC00901, ANRIL, and LIFR-AS1 contributes to PC progression [30], [31], [32], [33]. Innovative protocols for the isolation and analysis of tumor-derived fraction of circulating cell-free DNA (cfDNA), such as liquid biopsies, have an optimistic potential for early diagnosis of cancers via detecting methylations, point mutations, gene fusions, among other cancer-related signatures [34], [35], [36].

In the pharmacological context, several types of therapeutic approaches for PC have been explored over the last decade, such as poly-ADP-ribose polymerase (PARP) inhibitors (e.g., olaparib, veliparib, rucaparib, and talazoparib), mitogen/extracellular signal-related kinase (MEK) inhibitors, EGFR inhibitors, KRAS targeting agents, JAK/STAT inhibitors, hydroxychloroquine, immunotherapy, and electrochemotherapy. Nonetheless, the number of patients who may benefit from these therapies is constrained due to the particular attributes of the pancreatic tumor microenvironment, i.e., unique immune structure and cell-to-cell communication [37], [38], [39], [40]. Thus, the current leading treatment for advanced PC is surgery followed by adjuvant chemotherapy; however, solely a few patients are identified with locally resectable, non-metastatic illness [41,42]. Accordingly, the accurate comprehension of the epigenetic regulation of the molecular mechanisms underlying PC pathogenesis might represent a novel source of next-generation molecular medicine and diagnostics for this disease [43,44].

In this regard, miRNAs and lncRNAs have been projected as encouraging epigenetic clinical targets for PC [45], [46], [47], [48], [49], [50], [51]. Both miRNAs and lncRNAs can be found in specialized tissues [52,53] as well as in other types of biological samples (e.g., blood, plasma, serum, urine, exosomes, and stool), in the form of circulating miRNAs and lncRNAs [54], [55], [56], [57]. Significantly, exosomal miRNAs and lncRNAs are involved in homeostasis regulation because they function as mediators of cell-to-cell communication. Additionally, their altered expression can cause tissue dysfunction, aging, and a myriad of diseases [58]. It has been evidenced that these exosomal ncRNAs play a fundamental role in the regulation of a wide range of cancer-associated events, such as angiogenesis, metastasis, drug resistance, and immune escape [59]. Besides, since exosomal ncRNAs are highly stable and protected from enzymatic and chemical degradation by the membrane of exosomes, they are prospective biomarkers for different types of cancer [60], [61], [62]. Such is the case of exosomal miRNAs miR‑21 and miR‑210, which have been suggested as candidate biomarkers for the diagnosis of PC [63].

Therefore, the quantification of the expression levels of these ncRNAs in such samples leads to the identification of dysregulated (upregulated or downregulated) miRNAs and/or lncRNAs in multiple pathological conditions [64,65]. In fact, miRNAs and lncRNAs might serve as prognostic, diagnostic, and predictive biomarkers for PC [66]. Consistently, analyzing their expression profile in patients with suspected PC could be consolidated as a molecular diagnostic method in the near future. In addition, deciphering which miRNAs and lncRNAs are crucially implicated in PC development and progression might help to lay the groundwork for the development of ncRNA-based drugs aimed to re-establish the physiological levels of miRNAs and lncRNAs [67,68].

Given this, a variety of total RNA isolation protocols have been devised to investigate the physiological and pathological roles of miRNAs and lncRNAs. For example, early published protocol for miRNA isolation based on guanidine thiocyanate-phenol-chloroform extraction followed by RNA precipitation, commonly performed with TRIzol reagent (Thermo Fisher Scientific, Waltham, MA, USA) [69,70]. Nonetheless, high contamination levels could be present when using the aforesaid method and miRNAs with small guanine-cytosine content may be lost during phenol-chloroform extraction. As a consequence, more efficient procedures for small RNAs isolation have been conceived, which consist of placing the aqueous phase obtained during the phenol-chloroform extraction on an RNA adsorption column and, subsequently, wash and elute the RNA [69]. Two examples of well-known column-based miRNA isolation kits are: miRVana (Thermo Fisher Scientific) and miRNeasy (Qiagen, Hilden, Germany) [71].

Solid-phase isolation techniques have also been designed to separate miRNAs from clinical samples. The rationale for those protocols is based on the fact that miRNAs can be retained by suitable solid sorbents (e.g., fibers or membranes) [70]. Isolate II (Bioline, London, UK) and Norgen Total (Norgen Biotek, Thorold, ON, Canada) are representative examples of solid-phase-based total RNA isolation kits for miRNA extraction [71]. Similar to miRNA isolation protocol, both guanidine thiocyanate-phenol-chloroform and column-based systems are the mainstay protocols for lncRNA isolation. Despite this, lncRNA samples are often contaminated with organic and phenolic compounds when using TRIzol reagent, so column-based approaches are the most appropriate for lncRNA isolation [72,73]. Additionally, methods based on immunoprecipitation are frequently used to enrich lncRNAs linked to particular proteins [74]. At the end, miRNA and lncRNA expression profiling is performed using different techniques and tools, such as microarrays, qRT-PCR, Serial Analysis of Gene Expression (SAGE), RNA sequencing, and northern blots [75,76].

**Fig. 1:**
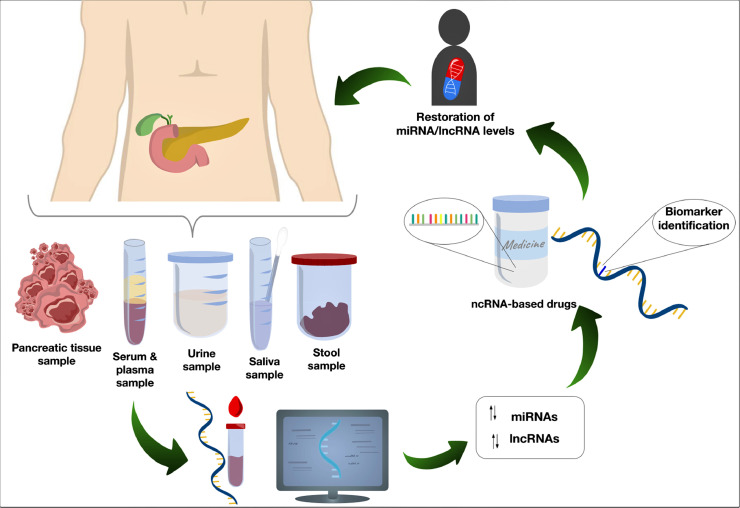
Potential clinical applications of miRNAs and lncRNAs. The analysis of diverse types of samples (e.g., PC tissues, serum, plasma, urine, saliva, and stool) can lead to the identification of dysregulated circulating miRNAs and lncRNAs involved in PC progression, which might set the foundations for creating ncRNA-based drugs and diagnostic protocols for PC.

The general overview of the design of miRNA- and lncRNA-based clinical approaches for PC is depicted in Fig. 1.

MiRNAs are short (20–24 nucleotides), endogenous, highly conserved RNA molecules that regulate gene expression post-transcriptionally. The discovery of miRNAs occurred in 1993 when Lee et al. [77] studied the regulatory mechanisms of LIN-14 protein expression in the nematode *Caenorhabditis elegans*. Subsequently, this finding led to the identification of miRNAs in a wide range of species, including *Homo sapiens, Mus musculus, Drosophila melanogaster*, and *Arabidopsis thaliana* [78], [79], [80]. Besides, over the last decades, the altered expression of miRNAs has been found to be associated with many multifactorial human diseases, such as neuropsychiatric disorders, neurodegenerative diseases, parasitic diseases, hair loss, diabetes, cancer, osteoporosis, COVID-19, pediatric diseases [81], [82], [83], [84], [85], [86], [87], [88], [89]. Further, these master regulators of gene expression have been demonstrated to be involved in cystic diseases, irritable bowel syndrome, smoking-induced chronic diseases, gastrointestinal cancers, hepatocellular carcinoma, and regulation of stem cell populations [90], [91], [92], [93], [94], [95]. As a matter of fact, let-7 family, miR-200 family, miR-21, miR-155, miR-27a, miR-205, miR-34a, miR-106a, miR-506, miR-23a/b, miR-216a, miR-29a/c, miR-221, miR-372, miR-212, miR-196b, miR-191, miR-182, miR-375, miR-142, miR-455, miR-15a, miR-202, miR-300, amongst other miRNAs, own a remarkable potential as both diagnostic and therapeutic tools for PC [20,45,96,97].

On the other hand, lncRNAs are a class of endogenic ncRNAs whose average size is longer than 200 nucleotides. Interestingly, lncRNAs can interact with miRNAs, mRNAs, DNA, and proteins, thus regulating gene expression at multiple levels, i.e., epigenetically, post-transcriptionally, translationally, and post-translationally [98,99]. Remarkably, the study of lncRNAs has allowed a more in-depth understanding of the etiological principles of a variety of genetic diseases since their discovery [100,101]. Indeed, since lncRNAs are implicated in a vast range of biological functions, their dysregulation has been related to different human pathologies, including ischemic stroke, cancer, viral diseases, diabetes, and neurodegenerative diseases [86,[102], [103], [104], [105], [106]]. Under this premise, relevant studies have demonstrated that several lncRNAs, such as HOTTIP, RP11–567G11.1, HOTAIR, MALAT-1, H19, GAS5, FEZF1-AS1, BANCR, LINC01111, TUG1, and DUXAP8 have a noteworthy potential as theragnostic targets for PC [46,[107], [108], [109]]. Further, aberrant ncRNA biogenesis are commonly found in human cancers due to mutations or dysregulations that affect the components of such pathways [76,110].

According to the previous information, miRNAs and lncRNAs own an emerging potential as biomarkers and therapeutic targets for PC. Therefore, throughout this review, we present a general overview concerning the most recent experimental evidence of the functional implications of miRNAs and lncRNAs within the molecular pathophysiology of PC to shed light on their clinical significance. Furthermore, we discuss several challenges that should be addressed in forthcoming studies to design innovative ncRNA-mediated medications for PC.

### Clinical implications of miRNAs in pancreatic cancer

PC is associated with multiple molecular pathways that stimulate its accelerated progression and intricate complexity. The inactivation of tumor suppressor genes, activation of oncogenes, and dysregulation of signaling pathways lead not only to the development of clinical symptoms [111], but also to the acquisition of chemoresistance in PC cells as well [112,113]. Particularly, studies have shown that miRNAs play a relevant role in pancreatic tumorigenesis since these RNA molecules regulate several cellular processes that comprise the expression and function of genes involved in cell proliferation, antitumor immune response, apoptosis, invasion, metastasis, and drug resistance [114]. Accordingly, the identification of abnormally expressed miRNAs related to PC pathological events could allow designing novel diagnostic protocols and medications for this disease. As a matter of fact, it has been stated that RNA-centered tools for prognostic, diagnostic, and predictive aims will experiment with remarkable growth in the following years, characterized by a forecasted investment of approximately 6.8 billion dollars by 2028 [115]. Even so, there are several challenges that must be resolved for these approaches to be successful in the future.

Firstly, in the case of miRNAs as biomarkers, preservation of miRNA purity and integrity represents a major constraint during the isolation and purification of these nucleic acids [116]. In the same sense, storage conditions and time must be precisely defined to prevent changes in miRNA composition before their analysis. As well, the source of the sample is another factor that should be considered when studying miRNA implications in cancer since miRNA expression varies between samples of the same patient (e.g., serum and plasma), hence, the implementation of data normalization with internal controls should be mandatory in these assays [116]. Secondly, in relation to the use of miRNAs as therapeutic agents, a number of hurdles, including efficient delivery systems, administration routes, effective dosages, off-target and immunostimulatory effects, and toxicity, must be aborded in forthcoming studies so that miRNA-based drugs for cancer can enter the pharmaceutical market [117,118].

#### miRNAs as PC biomarkers

To begin with, Zou et al. [119] investigated serum-derived exosomes and tissues from PC patients and noticed that let-7b-5p, miR-19a-3p, miR-19b-3p, miR-25–3p, miR-192–5p, and miR-223–3p were significantly upregulated in such samples. Similarly, the outcomes of another study elucidated that miR‑126‑3p, miR‑139‑5p, miR‑424‑5p, miR‑454‑3p, miR‑1271‑5p, miR‑3613‑5p, and miR‑5586‑5p were downregulated in tissues derived from patients with an early stage of PC. Besides, this miRNA signature might be applied to classify PC patients into low-risk and high-risk groups [120]. As a result, all these miRNAs are proposed as biomarkers for the timely identification of PC; however, more studies are required to confirm their reliability in PC diagnostic.

Later, miR-1290 and miR-1246 were found to be overexpressed in the serum of PC patients [121]; indeed, higher expression of miR-1290 was more frequent in patients at stage III and IV of PC, indicating that they were independent risk factors for PC and might be useful as biomarkers for PC diagnosis, along with the antigen CA19–9. Nonetheless, additional studies are required to validate the prognostic value of these miRNAs for the PC [121]. An investigation in which serum samples of PC patients were examined led to discover 13 PC signature miRNAs that can help to distinguish between PC patients and healthy individuals (i.e., miR-125a-3p, miR-125b-1–3p, miR-204–3p, miR-575, miR-1469, miR-4294, miR-4476, miR-4792, miR-6075, miR-6729–5p, miR-6820–5p, miR-6836–3p, and miR-6893–5p) [122]. The same study identified 432 serum miRNAs that could indicate whether a PC patient is operable (can go through surgery). Hence, these miRNAs profiles could be noteworthy to evaluate PC patients' surgical feasibility and develop better diagnoses for this type of cancer [122].

Likewise, Shams et al. [123] combined diverse serum expression profiles of miRNAs to identify the most relevant miRNA signatures for PC diagnosis. Subsequently, miR-92a-5p, miR-125a-3p, and miR-4530 were found to be the most significantly downregulated in PC. While, a substantial upregulation of miR-642b-3p, miR-663a, miR-1246, miR-1469, miR-5100, and miR-8073 was observed. Additional analysis revealed miR-125a-3p, miR-642b-3p, and miR-5100 as the most potent biomarkers for PC diagnosis. However, this dataset does not support the immediate clinical use of these biomarkers until further validation [123].

In addition, Lee et al. [124] analyzed serum samples of PC patients, reporting 39 circulating miRNAs as PC-specific diagnostic markers; among those, 15 miRNAs had already been reported as PC indicators in previous studies. qRT-PCR analysis revealed that miR-155–5p, miR-661, miR-4703–5p, and miR-7154–5p were significantly downregulated in the PC samples, whereas let-7b-5p, miR-22–3p, miR-4486, and miR-5100 were significantly upregulated; hence, these miRNAs may be applied for the early diagnosis of PC. Subsequently, a microarray-based analysis evidenced that hsa-miR-210 is significantly upregulated in PC, whereas hsa-miR-216a/b, hsa-miR-217, hsa-miR-375, and hsa-miR-634 are downregulated. Therefore, these miRNAs represent potential biomarkers for PC diagnosis [125].

Recently, Hata et al. [126] reported that miR-593–3p is significantly upregulated in peritoneal lavage fluids of patients with PC. Increased expression of this miRNA is correlated with the occurrence of micrometastasis even in patients with localized PC. Accordingly, miR-593–3p could be a promising prognostic indicator for PC patients subjected to staging laparoscopy.

#### miRNAs as therapeutic targets for PC

Interestingly, an investigation in which PC cells were treated with the chemotherapeutic drug doxorubicin displayed downregulation of miR-137 and autophagy induction, while induced overexpression of miR-137 promoted the therapeutic effect of doxorubicin activating cell apoptosis, diminishing cell survival, and blocking autophagy. The positive effect of miR-137 was linked to the fact that such miRNA regulates the expression of ATG5, a protein implicated in autophagy. Since autophagy may be related to chemoresistance, miR-137 could be used in combination with doxorubicin to treat PC [127]. In a similar study, He et al. [128] suggested that miR-137 is a prospective clinical target for PC since it reduces PC stemness and tumorigenicity by targeting KLF12 in human PC cell lines, thus inhibiting β-catenin nuclear translocation as well as Wnt signaling activation.

Fang et al. [129] detected that miR-106b, miR-125b, miR-148a, and miR-320a/c were upregulated in cancer-associated fibroblasts, while miR-29a, miR-378d, miR-422a, and miR-1285 were downregulated after treatment with gemcitabine (a chemotherapeutic agent for PC). They also demonstrated that miR-106b promotes gemcitabine resistance in PC via targeting TP53INP1, which is associated with oncogenesis and tumorigenesis. As a result, miR-106b could be a promising therapeutic target for gemcitabine resistance management in PC. In 2020, Wu et al. [130] suggested that the reduction of eIF5A2 expression to suppress autophagy and increase apoptosis in pancreatic ductal adenocarcinoma (PDAC) in vivo via plectin-1/miR-9 nanocomplexes greatly improves the anti-cancer impact of doxorubicin.

Additionally, Meng et al. [131] investigated the miRNA profile in gemcitabine resistance pancreatic ductal adenocarcinoma (PDCA), and their results revealed that miR-146a-5p was significantly downregulated in PDCA tissues. Further experiments showed that miR-146a-5p inhibited PDAC cell growth and made PDAC cells more susceptible to gemcitabine treatment by targeting TRAF6, a signal transducer involved in regulating inflammation and immunity. MiR-146a-5p was also shown to suppress the miR-146a-5p/TRAF6/NF-κB p65 axis, which regulates PDAC cell proliferation and chemoresistance [131]. Furthermore, Panebianco et al. [132] demonstrated that overexpression of miR-217 (downregulated in human PC) boosts PC sensitivity to gemcitabine by inhibiting cell cycle progression in PDAC cells, thus representing a remarkable therapeutic target for PC.

Liu et al. [133] noticed that miR-3662 is downregulated in PDAC cell lines and tissues. Besides, they also found that miR-3662 suppresses gemcitabine resistance and aerobic glycolysis in PDAC cells by regulating the expression of HIF-1α, which is related to chemoresistance and tumorigenesis. Hence, co-delivery of this miRNA with gemcitabine could be a promising approach to overcome PC gemcitabine resistance.

Xu and Zhang [134] detected that miR-299–3p is downregulated in PC cells and tissues. Mechanistically, the downregulation of this miRNA occurs due to the high levels of expression of TUG1, which functions as a molecular sponge of miR-299–3p. TUG1 is an oncogene in many types of cancer that promotes cell proliferation, invasion, migration, and epithelial-mesenchymal transition, and in PC tissues, its expression was negatively associated with miR-299–3p expression. Moreover, researchers noticed that the inhibition of the interaction TUG1/miR-299–3p repressed PC malignant progression by suppressing the Notch1 pathway, a highly conserved cell signaling system. Accordingly, these results imply that blocking the Notch1 pathway by repressing the TUG1/miR-299–3p axis might be a potential therapy option for PC [134].

To further elucidate the roles of miRNAs in PC, Wu et al. [135] inquire about the miRNA profile of PC-1.0 derived exosomes, revealing 62 upregulated ones. Remarkably, miR-125b-5p was shown to be substantially overexpressed in highly invasive PC cells, increasing migration, invasion, and epithelial-to-mesenchymal transition via targeting the tumor suppressor STARD13. Besides, the upregulation of miR-125b-5p was associated with MEK2/ERK2 signaling activation. These outcomes suggest that miR-125b-5p has a major role in PC metastasis. The results obtained in another inquiry supported that hypoxia upregulates the expression of miR-616–3p and miR-4465 in pancreatic stellate cells-derived exosomes. These miRNAs were implicated in the proliferation and invasion of PC cells by targeting PTEN, activating the AKT pathway [136].

In another recent investigation, Zhou et al. [137] isolated PC stem cells from xenograft cells and detected that miR-146b-3p was significantly downregulated in such specimens. Additionally, they demonstrated that miR-146b-3p targets a protein, namely MAP3K10, involved in tumorigenesis and the survival of PCs. Finally, they concluded that miR-146b-3p might induce apoptosis and repress proliferation in PC stem cells downregulating MAP3K10, and therefore it has an outstanding potentiality for the development of miRNA-based therapies for PC. To further illustrate the roles of miRNAs in PC, Chang et al. [138] analyzed M2 macrophage-derived extracellular vesicles and noticed that miR-21a-5p was substantially upregulated in such structures. In the same study, they elucidated that miR-21a-5p promotes the differentiation and activity of PC stem cells through targeting KLF3, a transcription factor with putative antitumor activity.

Compelling evidence obtained from another inquiry revealed that PC cell-derived exosomal miR-27a is linked with the angiogenesis of human microvascular endothelial cells via targeting BTG2, a protein involved in cell differentiation, apoptosis, antiproliferation, and DNA damage repair, and could be relevant for treating PC; nevertheless, further studies are required to fully understand the molecular crosstalk between this miRNA and angiogenic factors [139]. In a preclinical study, tumor xenograft-bearing mice were treated with a miR-24–3p mimics formulated within polymeric nanoparticles, and, as a consequence of the activation of necrosis and apoptosis, tumor inhibition was observed. Additionally, it was determined that the direct targets of miR-24–3p are PIM1 and PIM2, two proteins associated with oncogenesis [140].

**Fig. 2:**
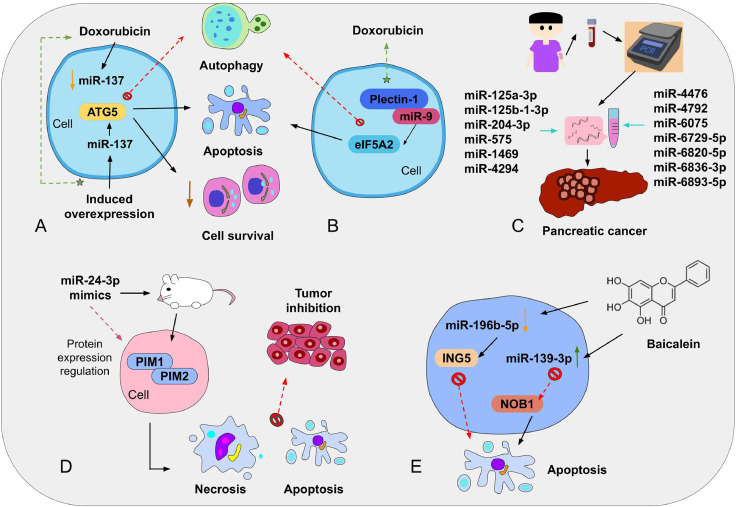
Schematic representation of important clinical applications of miRNAs in PC. (A) MiRNAs have been discovered to play an important role in cancer progression if combined with chemotherapy medicines, such as doxorubicin which, if used alone, can downregulate miR-137, triggering autophagy; while induced overexpression of miR-137 can promote doxorubicin effects, diminish cell survival, and block autophagy by regulating ATG5 expression. (B) Similarly, downregulating eIF5A2 expression via plectin-1/miR-9 complexes lead to autophagy suppression, increased apoptosis, and improvement of doxorubicin anti-cancer effects. (C) Serum samples of PC patients indicated that a specific miRNA signature conformed by 13 miRNAs might be helpful for PC diagnosis. (D) Preclinical studies in mice with miR-24–3p mimics showed that the targets of such miRNA are PIM1 and PIM2; its use resulted in cell apoptosis and necrosis. (E) Treatment with baicalein also affects miRNA expression, and miR-196b-5p and miR-139–3p were significantly affected by this flavonoid. Further assays evidenced that miR-139–3p, by inhibiting NOB1, stimulated apoptosis in PANC cells, while miR-196–5p suppressed this mechanism targeting ING5.

In another investigation, miR-145 was detected to be downregulated in PC tissues and cells. Consistently, induction of the expression of miR-145 in vivo disrupted tumor growth by suppressing the TGF-β signaling pathway (which promotes cell differentiation, cell proliferation, and chemotaxis) and inhibiting epithelial-mesenchymal transition. This evidence indicates that miR-145 is a possible candidate for anti-cancer drug development [141]. Besides, an investigation in which PC cells were treated with baicalein (an active flavonoid present in *Scutellaria baicalensis Georgi*) supported that this therapeutic approach enhances apoptosis and cell cycle arrest by altering the expression of at least 59 miRNAs, where the most significantly affected miRNAs are miR-196b-5p (downregulated) and miR-139–3p (upregulated) [142]. Additional experiments evidenced that miR-139–3p stimulates apoptosis of PANC-1 cells by inhibiting NOB1 expression, whereas miR-196b-5p can suppress the apoptosis mechanism targeting ING5. Therefore, baicalein might serve as an innovative therapy for PC; nevertheless, more research is required to unveil the underlying molecular mechanism of this flavonoid [142]. A few of these clinical applications of miRNAs in PC are illustrated in Fig. 2.

Long et al. [143] examined PC cells and tissue samples and detected that miR-409 was substantially downregulated. In fact, the decreased expression of this miRNA was linked with a poor survival rate of PC patients and tumor development. Thereafter, researchers evidenced that miR-409 might inhibit tumor progression by targeting GAB1, a protein with oncogenic implications in PC; however, additional tests are required to disclose the molecular interface and the theragnostic significance of miR-409 in PC. Similarly, Li et al. [144] noticed that miR-190b had been downregulated in PC cell lines and tissues. Additionally, they found that this miRNA targets factors involved in the malignant progression of PC (i.e., MEF2C and TCF4), and hence miR-190b is a prospective target for the diagnosis and management of PC. Very recently (in 2022), miR-802 was reported to attenuate KRAS-induced acinar-to-ductal metaplasia via suppressing SOX9 activity and F-actin reorganization, thus inhibiting PC initiation. Nonetheless, upcoming studies should validate the therapeutic use of miR-802 for PC [145]. Some of the most significant biological and clinical implications of miRNAs in PC are listed in Table 1.

**Table 1:** Biological and clinical implications of miRNAs in PC pathophysiology.

Clinical inference	miRNA	Regulation in PC	Target	Main conclusion	Refs.
PC diagnosis	let-7b-5p	Upregulated	–	Prospective serum biomarkers for PC early diagnosis	[119]
	miR-19a-3p	Upregulated	–		
	miR-19b-3p	Upregulated	–		
	miR-25–3p	Upregulated	–		
	miR-192–5p	Upregulated	–		
	miR-223–3p	Upregulated	–		
	miR‑126‑3p	Downregulated	–	Seven-miRNA signature that might help to classify early PC patients into high‑ and low‑risk groups	[120]
	miR‑139‑5p	Downregulated	–		
	miR‑424‑5p	Downregulated	–		
	miR‑454‑3p	Downregulated	–		
	miR‑1271‑5p	Downregulated	–		
	miR‑3613‑5p	Downregulated	–		
	miR‑5586‑5p	Downregulated	–		
	miR-1290	Upregulated	–	Prospective biomarkers for PC diagnosis	[121]
	miR-1246	Upregulated	–		
	miR-125a-3p	Downregulated	–	Prospective biomarkers for PC diagnosis	[123]
	miR-92a-5p	Downregulated	–		
	miR-4530	Downregulated	–		
	miR-642b-3p	Upregulated	–		
	miR-663a	Upregulated	–		
	miR-1246	Upregulated	–		
	miR-1469	Upregulated	–		
	miR-5100	Upregulated	–		
	miR-8073	Upregulated	–		
	miR-155–5p	Downregulated	–	Prospective biomarkers for PC diagnosis	[124]
	miR-661	Downregulated	–		
	miR-4703–5p	Downregulated	–		
	miR-7154–5p	Downregulated	–		
	let-7b-5p	Upregulated	–		
	miR-22–3p	Upregulated	–		
	miR-4486	Upregulated	–		
	miR-5100	Upregulated	–		
	miR-210	Upregulated	–	Prospective diagnostic and therapeutic biomarkers for PC	[125]
	miR-216a/b	Downregulated	–		
	miR-217	Downregulated	–		
	miR-375	Downregulated	–		
	miR-634	Downregulated	–		
	miR-593–3p	Upregulated	–	Prognostic indicator for PC patients subjected to staging laparoscopy	[126]
Therapeutic targets	miR-137	Downregulated	KLF12	miR-137 reduces PC stemness and inhibits Wnt/β-catenin signaling	[128]
	miR-106b	Upregulated	TP53INP1	miR-106b promotes gemcitabine resistance in PC	[129]
	miR-146a-5p	Downregulated	TRAF6	miR-146a-5p regulates gemcitabine resistance and PDAC cell growth	[131]
	miR-217	Downregulated	–	Induced miR-217 expression enhances gemcitabine sensitivity	[132]
	miR-3662	Downregulated	HIF-1α	Induced miR-3662 expression represses gemcitabine resistance and aerobic glycolysis	[133]
	miR-299–3p	Downregulated	–	TUG1/miR-299–3p axis is involved in malignant progression of PC	[134]
	miR-125b-5p	Upregulated	STARD13	miR-125b-5p promotes PC metastasis	[135]
	miR-616–3p	Upregulated	PTEN	miR-616–3p and miR-4465 promote PC progression and metastasis via repressing PTEN/AKT pathway	[136]
	miR-4465	Upregulated	PTEN		
	miR-146b-3p	Downregulated	MAP3K10	Induced miR-146b-3p expression inhibits PC stem cell proliferation and induces apoptosis	[137]
	miR-21a-5p	Upregulated in macrophage-derived extracellular vesicles	KLF3	miR-21a-5p enhances PC stem cell differentiation and activity	[138]
	miR-27a	Upregulated	BTG2	miR-27a enhances angiogenesis of human microvascular endothelial cells in PC	[139]
	miR-145	Downregulated	TGF-β signaling pathway	Induced miR-145 expression suppresses tumor growth and epithelial-mesenchymal transition in PC	[141]
	miR-409	Downregulated	GAB1	miR-409 is a prospective tumor suppressor of PC	[143]
	miR-190b	Downregulated	MEF2C and TCF4	Induced miR-190b inhibits malignant progression of PC	[144]

### Clinical inferences of lncRNAs in pancreatic cancer

LncRNAs are implicated in a wide range of molecular mechanisms related to the development of gastrointestinal cancers, such as resistance to apoptosis, chemoresistance, cell differentiation, division, migration, and invasion [146,147]. As a matter of fact, lncRNAs play crucial roles in cancer pathways since they act as biological sponges that modulate miRNA levels, thus affecting the regulation of tumor suppressors and oncogenes [148,149]. Under this premise, understanding the molecular crosstalk between lncRNAs and miRNAs might help pave the way for designing innovative ncRNA-centered therapies and diagnoses for PC.

#### lncRNAs as therapeutic targets for PC

Xu et al. [150] demonstrated that induced overexpression of the lncRNA DLEU2L (downregulated in PC tissues) hinders gemcitabine resistance via sponging miR-210–3p. Mechanistically, the suppressive effect of DLEU2L on miR-210–3p promotes the upregulation of BRCA2, thus stimulating apoptosis and repressing PC cell proliferation, migration, and invasion via blocking the Warburg effect (aerobic glycolysis) as well as AKT/mTOR signaling. Thereafter, in another study, the lncRNA ANRIL was detected to be overexpressed in PC tissues, depicting that ANRIL triggers HMGB1-induced cell autophagy (a pivotal process in oncogenesis) and augments resistance to gemcitabine via sponging miR-181a, the miRNA that targets HMGB1 protein [151].

Throughout an interesting investigation, the lncRNA HIF1A-AS1 was detected to be upregulated in gemcitabine-resistant PC cells. Systematically, this lncRNA promoted the interaction of serine/threonine kinase AKT with YB1, thus triggering the phosphorylation of the latter (pYB1) [152]. The outcomes of this study also indicated that the translation of HIF-1α was enhanced due to the recruitment of pYB1 to HIF-1α transcripts, which was mediated by HIF1A-AS1. As a result, the upregulation of HIF-1α promoted glycolysis and gemcitabine resistance in PC cells. Intriguingly, researchers noticed that HIF-1α enhances HIF1A-AS1 transcription as well. Therefore, the molecular interplay between HIF1A-AS1 and HIF-1α encloses an attractive clinical potential for targeting PC [152].

Additionally, SNHG7 has been reported as a substantially upregulated lncRNA in PC tissues. This lncRNA functions as a competing endogenous RNA to miR-342–3p (whose gene target is ID4) via sponging mechanisms, promoting PC cell proliferation and metastasis. Remarkably, SNHG7 knockdown repressed PC tumorigenesis *in vivo* [153]. An additional investigation revealed that the lncRNA DANCR (upregulated in PC cells and tissues) promotes tumor progression in PC since it acts as a sponge to miR-33b, positively regulating the expression of MMP16, a protein involved in PC cell migration and invasion [154].

Later, Cao and Zhou [155] studied the relationship of both lncRNA SNHG12 and miR-320b in PC, and their results showed an increased expression level of SNHG12 during the progression of PC with a proportional rate to that of PC cell invasion and cell growth. Mechanistically, miR-320b is repressed by the SNHG12’s absorbing effect. Since miR-320b is a negative regulator of TRIAP1 (protein implicated in apoptosis), as well as a suppression element in different carcinomas, the SNHG12-mediated downregulation of this miRNA stimulates epithelial-mesenchymal transition, proliferation, and invasion of PC cells [155]. These facts suggest that the effects of PC could be ameliorated via therapeutically silencing SNHG12. In contrast, lncRNA LINC00671 was noticed to be downregulated in PC patients and cell lines. Moreover, induced overexpression of LINC00671 was linked to the inhibition of cancer cell proliferation through the suppression of epithelial-mesenchymal transition, ERK, and AKT pathways [156].

Another lncRNA with promising medical implications in PC is LINC00857. Meng et al. [157] demonstrated that this lncRNA is upregulated in PC tissues and cells due to N^6^-Methyladenosine (m^6^A), which methylates LINC00857 and enhances its stability. As a consequence, the increased expression of LINC00857 foments the downregulation of miR-150–5p and upregulation of E2F3 by exerting a sponging effect on the former. Since E2F3 is an oncogene, its increased expression promotes PC tumorigenesis. Likewise, lncRNA TP73-AS1 was detected to be upregulated in PC cells and tissues and is linked with PC growth and metastasis since it promotes the expression of GOLM1 (a protein that participates in tumor progression) via downregulating miR-128–3p. Remarkably, TP73-AS1 silencing repressed tumor growth in vivo as well as PC cell proliferation, migration, and invasion in vitro, thus evidencing its therapeutic potential against PC [158].

In 2021, Liu et al. [159] found that GATA3-AS1 is substantially upregulated in PC tissues and cell lines. The effects of GATA3-AS1 knockdown were also investigated, resulting in an augment of apoptosis and inhibition of cancer cell viability, proliferation, and invasion. Moreover, a bioinformatic analysis demonstrated that GATA3-AS1 plays an important role in the downregulation of miR-30b-5p expression, implying that this lncRNA can act as a sponge to miR-30b-5p, thus leading to the release of miR-30b-5p targeted transcript: TEX10, which has carcinogenic roles. The GATA3-AS1/miR-30b-5p/TEX10 axis is believed to be related to Wnt/β-catenin signaling in PC cells [159]. In another study, lncRNA FGD5-AS1 was discovered to be upregulated in PC cells, enhancing both cancer cell proliferation and migration. Mechanically, FGD5-AS1 sponges miR-520a-3p, causing its low expression in PC cells. Since KIAA1522 is the target of such miRNA, low levels of miR-520–3p triggered the upregulation of this oncogene, which has been demonstrated to increase the tumorigenicity of various cancers (e.g., breast and lung cancers). Overall, this investigation might provide an RNA-based alternative to treat PC [160].

Subsequently, Xu et al. [161] found that lncRNA CERS6-AS1 is significantly overexpressed in PC tissues and cells. Consistently, CERS6-AS1 overexpression increased the expression of YWHGA via sponging miR-217 and enhancing cancer cell growth, proliferation, and invasion. In addition, researchers showed that YWHGA promotes the phosphorylation of RAF1, thus activating ERK signaling. These findings imply that the CERS6-AS1/miR-217/YWHGA/RAF1 axis is a promising medical target for the treatment of PC. Experimental evidence has also demonstrated that lncRNA NORAD (upregulated in PC cells and tissues) is a plausible therapeutic target for PC since it enables the expression of ANP32E via blocking the regulatory activity of miR-202–5p; therefore, enhancing self-renewal and proliferation of PC stem cells [162].

Additionally, Luo et al. [163] noticed that induced suppression of lncRNA LINC01094, which is usually overexpressed in PC, leads to the inhibition of metastasis and tumorigenesis in mouse xenografts and lessens both metastasis and proliferation of PC cells. They also clarified that this lncRNA acts as an endogenous sponge that downregulates miR-577, thus allowing the overexpression of LIN28B (the target of miR-577) and triggering the PI3K/AKT pathway, which in turn promotes PC progression [163]. Moreover, Zhang et al. [164] detected that the lncRNA FGD5‑AS1 is overexpressed in PC cell lines and tissues. FGD5‑AS1 was also related to cancer cell proliferation, migration, and invasion owing to the fact that it suppresses the regulatory activity of miR-577. Indeed, FGD5‑AS1-mediated downregulation of miR-577 alters the expression levels of β‑catenin, LRP6, cyclin D1, AXIN2, and c‑Myc, thus affecting the Wnt/β-catenin signaling pathway and contributing to the progression of PC.

Another study established that the expression of lncRNA PVT1 was increased under hypoxia in PC cell lines. Remarkably, HIF-1α transcription was demonstrated to be promoted by PVT1, while PVT1 requires HIF-1α expression for its efficient transcription and transcript stabilization. As well, it was noticed that PVT1 increases HIF-1α post-translationally [165]. The findings of this investigation also showed that patients with high levels of PVT1 and HIF-1α presented worst survival rates than those overexpressing only one of the two. In addition, PVT1 knockdown impeded HIF-1α-mediated PC tumorigenesis. Therefore, the positive feedback loop PVT1-HIF-1α should be thoroughly examined in the future to design next-generation therapeutics for PC [165].

In 2021, Zhu et al. [166] showed that the lncRNA CRNDE is significantly upregulated in PC cells and tissues. Furthermore, they concluded that the overexpression of this lncRNA boosts the progression and angiogenesis of PC by sponging miR-451a, thus allowing the enhanced expression of protein CDKN2D implicated in the regulation of tumor growth. Accordingly, CRNDE-mediated modulation of the miR-451a/CDKN2D axis could be a reliable clinical target for PC. An additional study suggested that the lncRNA MIR99AHG, whose transcription is enhanced by FOXA1, sponges miR-3129–5p and recruits ELAVL1. As a result, MIR99AHG increases the progression of PC by regulating NOTCH2 expression and promoting the activation of the Notch signaling pathway [167]. Meng et al. [168] revealed that the lncRNA LINC01320 is upregulated in PC cell lines and sponges miR-324–3p, thus promoting PC cell growth and migration. However, LINC01320 downregulation represses the growth and migration of PC cells and mediates apoptosis, suggesting that such lncRNA may be a prospective target for PC therapy. The main results regarding the clinical and pathological implications of lncRNAs in PC are summarized in Table 2.

**Table 2:** Compelling evidence regarding the roles of lncRNAs in PC development and progression.

lncRNA	Regulation in PC	Targets	Main conclusion	Refs.
DLEU2L	Downregulated	miR-210–3p/ BRCA2	DLEU2L overexpression represses gemcitabine resistance and PC	[150]
ANRIL	Upregulated	miR-181a/ HMGB1	ANRIL triggers HMGB1-induced cell autophagy and promotes gemcitabine resistance	[151]
HIF1A-AS1	Upregulated	AKT/YB1/HIF-1α pathway	HIF1A-AS1 promotes glycolysis and gemcitabine resistance	[152]
SNHG7	Upregulated	miR-342–3p/ID4	SNHG7 enhances PC proliferation, migration, and invasion	[153]
DANCR	Upregulated	miRNA-33b/ MMP16	DANCR enhances PC proliferation and metastasis	[154]
SNHG12	Upregulated	miR-320b	SNHG12 promotes PC proliferation, invasion and epithelial-mesenchymal transition	[155]
LINC00671	Downregulated	Epithelial-mesenchymal transition, ERK, and AKT pathways	LINC00671 overexpression inhibits PC proliferation and metastasis	[156]
LINC00857	Upregulated	miR-150–5p/E2F3	Methylated LINC00857 enhances PC tumorigenesis	[157]
TP73-AS1	Upregulated	miR-128–3p/ GOLM1	TP73-AS1 enhances PC growth and progression	[158]
GATA3-AS1	Upregulated	miR-30b-5p/TEX10	GATA3-AS1 regulates apoptosis, proliferation, invasion, and stemness in PC	[159]
FGD5-AS1	Upregulated	miR-520a-3p/KIAA1522	FGD5-AS1 enhances PC proliferation and migration	[160]
CERS6-AS1	Upregulated	miR-217/YWHGA/RAF1	CERS6-AS1 enhances PC proliferation and metastasis	[161]
NORAD	Upregulated	miR-202–5p/ ANP32E	NORAD enhances PC proliferation and stem cell self-renewal	[162]
LINC01094	Upregulated	miR-577/LIN28B	LINC01094 enhances PC progression and PI3K/AKT pathway activation	[163]
FGD5‑AS1	Upregulated	miR‑577	FGD5‑AS1 activates Wnt/β‑catenin pathway and enhances PC proliferation, migration, and invasion	[164]
PVT1	Upregulated	HIF-1α	PVT1 knockdown suppresses HIF-1α-induced PC proliferation, migration, and invasion	[165]
CRNDE	Upregulated	miR-451a/CDKN2D	CRNDE enhances PC progression and angiogenesis	[166]
MIR99AHG	Upregulated	miR-3129–5p/ELAVL1/NOTCH2	MIR99AHG enhances PC progression via activating Notch signaling pathway	[167]
LINC01320	Upregulated	miR-324–3p	LINC01320 downregulation suppresses PC growth and migration	[168]

## Conclusions

Over the last few years, a number of relevant studies have elucidated the crucial epigenetic regulatory role of both miRNAs and lncRNAs in the pathophysiology of PC. In fact, it has been evidenced that the altered expression of these ncRNAs affects a variety of biological processes implicated in PC development and progression, such as apoptosis, autophagy, tumor growth, tumor suppression, chemoresistance, cancer cell proliferation, migration, and invasion. Moreover, miRNAs and lncRNAs are suggested as prospective biomarkers for accurate PC diagnosis and prognosis. Therefore, the experimental evidence conferred in this current review implies that miRNAs and lncRNA own a noteworthy clinical potential against PC. Nevertheless, it is worth mentioning that further investigations are required to properly understand the regulatory functions of miRNA and lncRNA transcriptomes that remain elusive in the etiology of PC.

### Future directions

As stated throughout the previous sections, molecular biologists have been assiduously analyzing the biological implications of miRNAs and lncRNAs in PC pathophysiology. Nonetheless, there are still a number of concerns and subtle questions that should be addressed in forthcoming investigations. For instance, different reports have suggested that melatonin might have an important modulatory role in the progression of PC since this indoleamine could induce cancer cell apoptosis via regulating the activity of a variety of pathways, such as vascular endothelial growth factor, oxidative stress, and heat shock proteins [169]. Moreover, the therapeutic role of melatonin in various types of cancers, including breast, oral, gastric, colorectal, and prostate cancer, has been linked with the regulation of the expression of certain miRNAs (e.g., let-7i-3p, miR-21, miR-24, miR-155, miR-34b-5p, miR-319, miR-148a-3p, miR-3195, and miR-374b) [170,171]. In the same context, relevant reports have shown that lncRNAs H19, MEG3, CPS1-IT1, and lnc010561 also display biological interactions with melatonin in cancer which are related to apoptosis, pyroptosis, and metastasis [172]. Therefore, more research is required to unveil the molecular crosstalk between miRNAs, lncRNAs, and melatonin, as well as their clinical significance in PC.

Besides, it is worth mentioning that diverse signaling pathways regulated by lncRNAs (e.g., DLX6-AS1, LINC00261, TSLNC8, SNHG1, LINC01133, LINC00462, DLEU2, PVT1, and H19) are implicated in PC progression. Some examples of these pathways are Wnt/β-catenin, NOTCH, TGFβ/SMAD, and JAK/STAT [173]. Similarly, miR-96, miR-193b, miR-206, miR-20a, miR-216a, miR-744, miR-940, miR-296, miR-615–5p, miR-301–3p, and miR-421 have been associated with JAK/STAT, MAPK/ERK, Wnt/β-catenin, TGFβ, and AKT/mTOR pathways [112]. Accordingly, future investigations should focus on continuing to illuminate the regulatory implications of the lncRNA and miRNA transcriptomes in the aforesaid PC-related pathways, especially those poorly studied in this research field, i.e., JAK/STAT and TGFβ/SMAD.

On the other hand, since glycolysis enhances PC progression, invasion, metastasis, epithelial-mesenchymal transition, angiogenesis, metastatic colonization of remote organs, and chemoresistance [174,175], the development of ncRNA-based approaches for glycolysis regulation has been suggested as a promising alternative for PC suppression [176]. In this regard, studying the modulatory effects of miRNAs and lncRNAs on those glycolytic proteins linked with cancer progression, e.g., HK1, GAPDH, PKM2, GLUT1, GLUT3, HIF-1α, CAV1, Ras, HK2, LDHA, PGI/AMF, and PFKFB3 [174,175,177], might be very favorable for the design of novel treatments for PC. Some examples of miRNAs and lncRNAs that have been recently associated with glycolysis regulation in PC are miR-210–5p, miR-202, miR-135, miR-505, BLACAT1, LINC00941, MIR210HG, and LINC01448 [178], [179], [180], [181], [182], [183], [184].

Since chemotherapy is one of the standard treatments for PC, chemoresistance is another important concern implicated in this disease [42,185]. This type of drug resistance is very common in PC and is linked with genetic or epigenetic alterations, desmoplastic stroma, metabolic reprogramming, tumor microenvironment, and epithelial-mesenchymal transition [186]. Under this premise, miRNAs and lncRNAs could be reliable tools for better management of PC chemoresistance. For instance, miR-210, miR-124, miR-7, miR-205, miR-21, miR-221, miR-200b/c, miR-155, among others, have been correlated with chemoresistance, whereas miR-153, let-7a, miR-33a, miR-205–5p, miR-138–5p, miR-203, and additional miRNAs have been demonstrated to be implicated in chemosensitivity [187]. Similarly, CASC2, GAS5, HCP5, HOST2, HOTTIP, HOTAIR, MEG3, PVT1, ROR, SNHG8, and TUG1 are examples of lncRNAs involved in the regulation of drug resistance in PC [188]. Consistently, these ncRNAs should be studied in-depth to generate next-generation medications that could be co-delivered with chemotherapeutic drugs for PC.

**Fig. 3:**
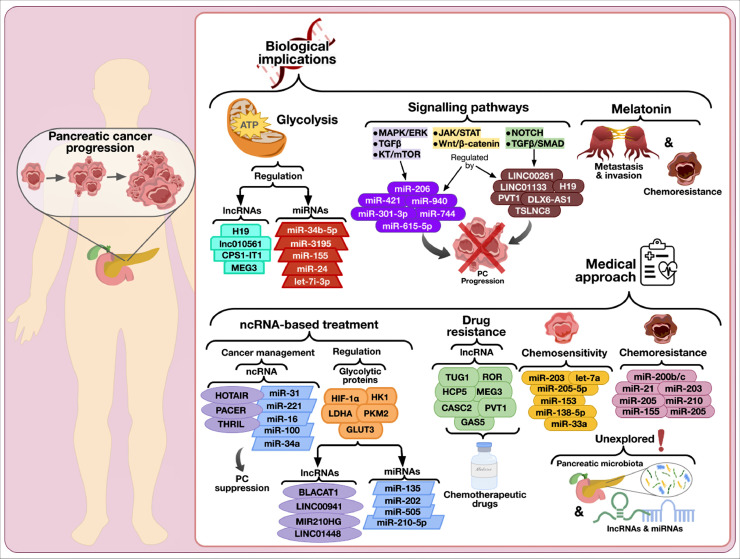
Future perspectives regarding the biological implications and medical approaches of miRNAs and lncRNAs in PC. As depicted, future research should focus on unveiling the regulatory roles of miRNAs and lncRNAs on glycolysis, PC-related pathways, melatonin, chemosensitivity, and chemoresistance. Likewise, it is necessary to explore the molecular crosstalk between pancreatic microbiota, miRNAs, and lncRNAs. As a whole, addressing these concerns could benefit the design of ncRNA-based treatments for PC.

In addition, even though the pancreas does not have local microbiota, dysbiosis (alteration of gut microbiota balance) and intestinal bacteria overgrowth promote a leaky gut, which in turn facilitates the translocation of intestinal microbiota into the pancreas [189]. Many studies have indicated that host microbiota could play an important role in PC [190,191]. As a matter of fact, experts have suggested that microbiota could contribute to PC progression by triggering inflammatory pathways involved in carcinogenesis, overturning both innate and adaptative immune responses, mediating chemoresistance, and interacting with other factors, including food and diet, bile acids, and tumor microenvironment [192,193]. Nevertheless, to the best of our knowledge, the interplay between pancreas microbiota, miRNAs, and lncRNAs in PC remains unexplored; thus, assessing these matters in upcoming studies could be beneficial for the design of new medicines for this ailment (Fig. 3).

It is worth mentioning that the identification of differentially expressed miRNAs during the different stages of PC could be helpful to overcome its progression [20]. Nonetheless, studies focused on this aim are still scarce. For instance, in 2015, Rachagani et al. [194] used a Kras^G12D^; Pdx1-Cre mouse model to examine the miRNA expression level from the precursor lesions to the final stage of PC (which is PDAC), and they noticed that a wide range of miRNAs had different expression levels at 10, 30, 40, and 50 weeks of PC progression. In fact, miR-216 and miR-217 levels reduced progressively in the mice models, whereas the expression levels of miR-21, miR-34c, miR-146b, miR-205, and miR-223 increased substantially. Hence, upcoming studies should be focused on unveiling the biological impact of those miRNAs and lncRNAs that are differentially expressed during PC development.

Intriguingly, in the past years, several RNA therapeutic agents (e.g., fomivirsen, mipomersen, eteplirsen, pegaptanib, patisiran, lumasiran, and givosiran) have been approved for medical use by the FDA [195,196]. As a result, investors are paying much attention to this research field, and various biopharmaceutical corporations have arisen, intending to develop ncRNA-based drugs. Some of these companies are Regulus Therapeutics, miRagen Therapeutics Inc., Mirna Therapeutics Inc., EnGeneIC, Santaris Pharma, and InteRNA Technologies, which are conducting miRNA-centered programs for a variety of human ailments, including cancer [117,197,198]. Further, the increasing interest in ncRNA-mediated treatments for human diseases has led to the initiation of a variety of ongoing clinical trials in which the prospective use of miRNAs and lncRNAs is being analyzed for cancer management.

Some of the most representative examples of these ncRNA molecules currently in clinical trials are miR-31 and miR-210 (oral cancer), miR-34a (melanoma, primary liver cancer, multiple myeloma, lymphoma, among other cancers), miR-100 (breast cancer), miR-155 (bladder cancer), miR-16 (non-small cell lung cancer and malignant pleural mesothelioma), miR-221 and miR-222 (hepatocellular carcinoma), THRIL and PACER (stomach cancer), and HOTAIR (thyroid cancer) [199]. To date, only one clinical trial is ongoing related to the application of a specific miRNA in PC detection (NCT03432624); such a study is centered on evaluating the use of miR-25 in the diagnosis of PC with a detection kit, but there is still no miRNA or lncRNA drug in clinical tests [48,200]. Thus, more comprehensive studies are needed to apply miRNA and lncRNA in PC theranostics.

Under such assertions, many challenges should be aborded in future studies for miRNA- and lncRNA-mediated therapeutics to reach the pharmacological breakthrough. For example, it has been established that ncRNA expression is affected by age, sex, body mass, physical activity, smoking, alcohol consumption, and diet [201,202], and hence these factors should be taken into account when developing ncRNA-based drugs and diagnoses. As well, toxicity analyses, improved delivery systems, reduction of the immunostimulatory potentiality of synthetic RNA medications, enhancement of on-target specificity, and lack of undesired on-target and off-target effects are concerns that must be considered to develop ncRNA-centered therapeutics [203].

In conclusion, from our personal perspective and based on our current knowledge on ncRNA-focused therapeutics for diverse human diseases, we assume that, over the coming decades, both miRNA- and lncRNA-based cancer therapeutics will reach sky-high. Relevantly, the development of mRNA vaccines for COVID-19 during the past two years has provided a crystal-clear demonstration that next-generation RNA-centered molecular medicine could be a significant lifesaver. In fact, as these types of treatments gain more attention, more opportunities will come up to extrapolate their application to various health issues. Although further research on the mechanisms and behaviors of ncRNA-based drugs within the human system is required, we believe that the information presented in this review will strengthen this research arena to set up the ncRNA-focused therapeutic pipeline for PC.

## Author contributions

LABV, SujayP conceived, performed the literature search, and wrote the manuscript. NFR, AGRD, SMOP, and HRZC performed the literature search and contributed to writing the manuscript. SP, AntaraB, AB, AKD critically revised the manuscript. All authors have reviewed and approved the final manuscript.

## Declaration of Competing Interest

The authors declare that they have no known competing financial interests or personal relationships that could have appeared to influence the work reported in this paper.
